# Exercise improves mental health status of young adults *via* attenuating inflammation factors but modalities matter

**DOI:** 10.3389/fpsyt.2022.1067890

**Published:** 2022-12-14

**Authors:** Jianxiu Liu, Yao Zhang, Xingtian Li, Dizhi Wang, Bolan Shi, Yanwei You, Leizi Min, Bicheng Luo, Yanchun Li, Qian Di, Xindong Ma

**Affiliations:** ^1^Vanke School of Public Health, Tsinghua University, Beijing, China; ^2^Division of Sports Science and Physical Education, Tsinghua University, Beijing, China; ^3^Soochow College, Soochow University, Suzhou, China; ^4^China Athletics College, Beijing Sport University, Beijing, China; ^5^China Academy of Sports and Health, Beijing Sport University, Beijing, China; ^6^Institute for Healthy China, Tsinghua University, Beijing, China; ^7^IDG/McGovern Institute for Brain Research, Tsinghua University, Beijing, China

**Keywords:** high-intensity interval training, moderate-to-vigorous intensity continuous training, mental health, immune inflammation, young adults, randomized controlled trial

## Abstract

**Introduction:**

The mental health of young adults is a global public health challenge. Numerous studies have demonstrated that exercise benefits mental health. However, it is still unclear which exercise mode is optimal for protecting mental health and its association with the immune system. This study aimed to compare the intervention effect of high-intensity interval training (HIIT) and moderate-to-vigorous intensity continuous training (MVCT) on mental health and assess the underlying mechanism of exercise interventions to improve the immune system, which facilitated the mental health status.

**Methods:**

This is a double-blinded RCT study conducted from October 13, 2020 to January 25, 2021 (ClinicalTrials.gov identifier: NCT04830059). Ninety-three participants who met the inclusion criteria were randomized into the HIIT (*N* = 33), MVCT (*N* = 32), and control groups (*N* = 28) with a mean age of 25.26 (SD = 2.21), and 43% of males enrolled in the study. Professional coaches guided participants in HIIT and MVCT groups to perform 40 min of exercise training three times a week for 12-week while those in the control group received 1 h of health education twice a week. Questionnaires related to mental health status and blood samples of inflammatory factors, including immunoglobulin A (IgA), immunoglobulin M (IgM), albumin (Alb), globulin (GLO), lymphocytes (LYM), and lymphocyte percentage (LYM) were assessed before and after the intervention.

**Results:**

We found that blood inflammation factors increased significantly in the control group during 12 weeks (ΔIgA = 0.16 g/L, ΔIgM = 0.092 g/L, ΔAlb = 2.59 g/L, ΔGlo = 3.08 g/L, ΔLYM = 0.36, and ΔLYM% = 3.72%, *p* < 0.05), and both MVCT and HIIT intervention could effectively defend the increased inflammatory response compared with the control group (IgA: MVCT β = −0.14, *p* < 0.001, HIIT β = −0.096, *p* < 0.05; IgM: MVCT β = −0.12, *p* < 0.001; HIIT β = −0.068, *p* < 0.05; Alb: MVCT β = −1.64, *p* < 0.05, HIIT β = −1.14, *p* > 0.05; Glo: MVCT β = −3.17, *p* < 0.001, HIIT β = −2.07, *p* < 0.01; LYM: MVCT β = −0.34, *p* < 0.05, HIIT β = −0.35, *p* < 0.05). However, the MVCT intervention modality was more conducive to enhancing positive affect (β = 0.52, *p* = 0.018) and well-being (β = 1.08, *p* = 0.035) than HIIT. Furthermore, decreased IgA, Alb, and Glo were associated with improved mental health.

**Conclusion:**

Both 12-week HIIT and MVCT are beneficial to the immune system. The MVCT intervention mode is recommended to prevent mental health problems and attenuate immune inflammation, and the immune system is a potential mechanism that exercises improving mental health.

**Clinical trial registration:**

[ClinicalTrials.gov], identifier [NCT04830059].

## 1 Introduction

Mental health was defined by WHO as a state of mental well-being in which the individual can perceive his/her abilities, face the stresses of their life, and work productively to contribute to society (1). Young adults are faced with rapid changes and high pressure in physiology, psychology, and society, which may potentially affect their physical and mental health. The mental health of young adults is a global public health challenge since mental disorders, such as depression, anxiety, stress, and post-traumatic stress, have accounted for a large proportion of their disease burdens (2). Mental health problems related to difficulty functioning, poor physical health, and even development concerns were recognized as systemic illnesses with a range of mechanisms, including immune and inflammatory pathways (3). The emergence of symptom sequelae, even below the diagnostic threshold, signals an increased vulnerability to life course–persistent mental health problems and consequences if not addressed early (4).

Many studies examined the effect of different modalities of exercise, including high-intensity interval training (HIIT) and moderate-to-vigorous intensity continuous training (MVCT), on mental health among healthy adults and mental disorder patients. Previous studies found that physical exercise has a positive relationship with subjective well-being in young adults, and this relationship differs by the intensity of exercise (5). Both low and moderate exercise intensity may work best to alleviate depressive symptoms and perceived stress among university students after a few weeks of intervention (6). Evidence also reported that moderate and vigorous intensity of exercise engaged in multiple times per week over eight or more weeks would potentially treat depression in adolescents and young adults (7). MVCT was also proven to alleviate depressive symptoms in adults with major depression (8). However, a recent study found that nine-week HIIT didn’t change the anxiety and depression symptoms, as well as objective markers of inflammation compared with the placebo-exercise group, indicating that nine-week HIIT may not be an optimal exercise mode for managing symptoms of anxiety or depression (9). Therefore, further intervention study is needed to design a longer intervention time to examine more recommended exercise modes for mental health.

The physiological and biochemical mechanisms of exercise on mental health include increased endorphins (10), improved mitochondria function (11), neurotransmitters (e.g., serotonin, dopamine, and noradrenalin) (12), the hypothalamic-pituitary-adrenal axis (13), and the immune pathway through the inflammation factors (14). Chronic inflammation could be attributed to the pathogenesis of mental health problems (15). Elevated inflammatory signaling dysregulates neurotransmitter metabolism and alters the neural activity of brain regions related to mood and emotion (16, 17). Peripherally released cytokines send signals via molecular, cellular, and neural routes, ultimately reaching the brain and enhancing central nervous system (CNS) inflammation (18). In general, heightened inflammation characterizes a series of disorders and systemic diseases, including cardiovascular disease, metabolic syndrome, rheumatoid arthritis, multiple sclerosis, and asthma, and each of these also features an elevated risk for depression (17, 19). Therefore, the immune inflammation system is essential in stimulating the body’s immune function through exercise, thus preventing diseases, including mental disorders. Previous studies reported that the level of immunoglobulin provided key information on the humoral immune status, and low levels of immunoglobulin A (IgA) and immunoglobulin M (IgM) define some humoral immunodeficiencies (20). Albumin (ALB) and globulin (GLO) are indispensable components in serum protein, which has been known as an important marker of chronic inflammation (21). Regular exercise can reduce the damage of unhealthy behavior to the immune function of the body in order to maintain immune homeostasis. Evidence reported exercise could increase the levels of circulating adrenaline, which could decrease pro-inflammatory cytokine production by monocytes and lymphocytes (LYM) (22). However, until now, few studies have compared the effect of HIIT and MVCT on mental health and change of the inflammatory factors, which potentially reflect the immune mechanism of exercise influencing young adults’ mental health.

Thus, in this study, we tried to answer which exercise intervention mode was more suitable for preventing mental health problems and attenuating immune inflammation. Especially, we conducted a double-blinded randomized controlled trial (RCT) to (1) investigate the intervention effect of HIIT and MVCT on mental health; (2) compare the effect of HIIT and MVCT on biochemical blood indicators related to inflammatory immune factors; (3) assessed the underlying mechanism of exercise intervention improved the immune system, which facilitated the mental health status. According to the previous articles (6–9), the research hypothesized that both HIIT and MVCT could improve the inflammation immune system. Moreover, MVCT would be better at improving the inflammatory factors associated with mental health status. This study could provide first-hand evidence of the effect of 12-week HIIT and MVCT intervention on mental health among young adults and have important implications for public health to prevent mental disorders.

## 2 Methods

### 2.1 Study design and population

This double-blinded RCT study started from October 13, 2020 to January 25, 2021 (ClinicalTrials.gov identifier: NCT04830059). One hundred healthy college students were recruited, and 93 participants who met the inclusion criteria were randomized into HIIT, MVCT, and control groups. We conducted the double-blind RCT using randomization, assignment concealment and blinding methods. Ninety-three participants remained after screening according to the inclusion criteria. Firstly, each participant was given an identification number. They were divided into different groups according to the simple random procedure (random number generated by computer) conducted by a staff not involved in measurement and data analysis. All participants were assigned to a certain group with the same probability. Secondly, the group information corresponding to each name and ID was hidden in an opaque envelope. The group was assigned confidentially before allocation to ensure the successful implementation of randomization. All subjects opened the envelopes in turn after completing the baseline assessment. The assignment hiding was performed by one researcher who was different from the researcher who conducted randomization. Thirdly, the participants in different intervention groups came to the fitness center on different days, so they didn’t know about the intervention program of the other groups. Participants and evaluators were blinded to group allocation and intervention assignments throughout the study.

**FIGURE 1:**
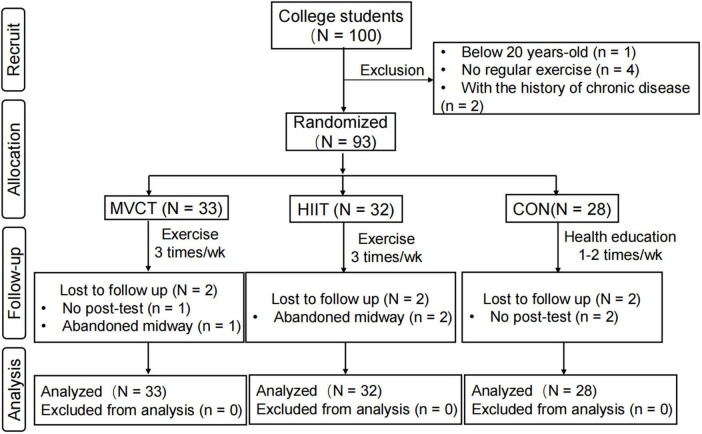
The CONSORT flow diagram. MVCT, moderate-to-vigorous intensity continuous training; HIIT, high-intensity interval training; CON, control.

The inclusion criteria of participants in this study were (1) healthy young college students with normal BMI aged from 20 to 30 years old; (2) without a history of chronic diseases or psychiatric disorders; (3) no regular exercise habits according to the screening of the international physical activity questionnaire; (4) without physical limitations (e.g., restricting injuries of the musculoskeletal system such as osteoarthritis) according to the widely used Physical Activity Readiness Questionnaire (PAR-Q) (23). The trial profile of the RCT study is shown in Figure 1. Professional coaches guided participants in HIIT and MVCT groups to perform 40 min of exercise training thrice a week for 12-week. Participants in the control group received 1 h of health education twice a week. The Institutional Review Board of Tsinghua University approved this study (IRB 20190091). All participants signed informed consent at the time of recruitment.

### 2.2 Process and interventions

#### 2.2.1 Study procedure

**FIGURE 2:**
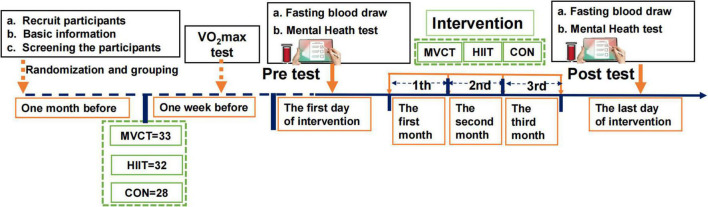
Experiment process of the RCT study. MVCT, moderate-to-vigorous intensity continuous training; HIIT, high-intensity interval training; CON, control.

Mental health and blood biochemical outcomes were measured before (baseline collected from October 13, 2020) and after exercise interventions (repeatedly measured from January 25, 2021). Participants completed all measurement items in the morning to avoid interference caused by different measurement times. 12-h fasted blood samples were obtained between 8:00 to 10:00 am. Immediately after blood collection, samples were cooled on ice for 30 min and centrifuged at 4000 rpm at 4°C for 10 min. Then collect the supernatant to obtain serum, and store the collected serum in the refrigerator at −80°C until analysis. The experiment process of the RCT study is described in Figure 2.

#### 2.2.2 Intervention

The intensity of HIIT and MVCT interventions was determined by the participants’ maximum oxygen uptake (VO_2max_), defined as the oxygen intake during an exercise intensity when actual oxygen intake reaches a maximum that can’t be improved by any effort (24). We measured VO_2max_ one week before the formal experiment by Bruce’s treadmill protocol, with oxygen consumption and ventilation monitored using Cosmed Fitmate metabolic system (Cosmed, Rome, Italy) (25). The participant ran on the treadmill at a fixed speed, which gradually increased until they met the following criteria to terminate: (1) the participants had the following symptoms, including dyspnea, cyanosis, dizziness, tinnitus, nausea, chest pain, extreme fatigue, painful expression, pale face, body shaking, etc.; (2) abnormal blood pressure (blood pressure 200/110mmHg, or diastolic blood pressure reduced by more than 20mmHg); (3) arrhythmia; (4) Rating of Perceived Exertion (RPE) ≥ 19; (5) The respiratory quotient is greater than or equal to 1.1 (6) they could not continue to run, and propose to stop the test; (7) unable to maintain the required speed for 10s. (26). Heart rate, expiratory and inspiratory volume were recorded during running. There were two professional coaches and research assistants to protect the safety of the participants during the test of maximum oxygen uptake.

After measuring VO_2max_, we developed the HIIT and MVCT exercise training intensity for each participant according to their individual VO_2max_. Referring to previous studies (20, 27, 28), the participants in the MVCT group were instructed to run on the treadmill for 20 min with a speed corresponding to 70%-75% of their VO_2max,_ added with a 10-min worm-up before the training and a 10-min cool-down after the training. The participants in the HIIT group were instructed to run on the treadmill for 1 min with a speed corresponding to 100% of the individual VO_2max_, followed by another minute of running with a speed corresponding to 50% of the individual VO_2max_, and repeated ten times for 20 min totally, added with a 10-min worm-up before the training and a 10-min cool-down after the training. A heart rate band (Polar H10, Finland) worn on the chest was used to detect the heart rate to monitor the real-time running speed. Before and after the 20-min exercise of HIIT and MVCT, participants performed 20 min of warm-up and cool-down, making the total intervention time 40 min. The participants in the control group received health education lessons for 1 h twice a week. Both the participants in HIIT and MVCT groups visited the fitness center three times a week. No participant was injured since we have professional coaches to guide and supervise them to exercise. Detailed information on the intervention program is shown in Table 1.

**TABLE 1:** The detail information of the training program.

Groups	Periods	Total time for one session	Frequency of the intervention	Training programs
MVCT	12 weeks	40-min	Three times/week	The participants in the MVCT group were instructed to run on the treadmill for 20 min with a speed corresponding to 70–75% of their VO_2max_, added with a 10-min worm-up before the training and a 10-min cool-down after the training.
HIIT	12 weeks	40-min	Three times/week	The participants in the HIIT group were instructed to run on the treadmill for 1 min with a speed corresponding to 100% of the individual VO_2max_, followed by another minute of running with a speed corresponding to 50% of the individual VO2max, and repeated ten times for 20 min totally, added with a 10-min worm-up before the training and a 10-min cool-down after the training.
Control	12 weeks	1 h	Two times/week	The participants were received health education lessons, which the main content including: (1) body posture assessment; (2) adjustment of body posture; (3) the method of stretching and relaxation; (4) the evaluation of body composition; (5) diet and health; (6) Sleep and health, etc.

### 2.3 Measurements and outcomes

#### 2.3.1 Mental health

We used subjective well-being as the metric of mental health, which consisting life satisfaction, positive affect, and negative affect. The score of subjective well-being is the sum of the standard scores of life satisfaction and positive emotions and then subtracted by the standard score of negative emotions.

##### Life satisfaction

Life satisfaction was measured by the Satisfaction with Life Scale (SWLS). Previous studies have demonstrated satisfactory reliability and validity of SWLS for Chinese college students (21). The SWLS contains five items, including: (1) “In most ways, my life is close to my ideal,” (2) “The conditions of my life are excellent,” (3) “I am satisfied with life,” (4) “So far I have gotten the important things I want in life,” and (5) “If I could live my life over, I would change almost nothing.” Respondents were asked to rate the extent to which they agree with or disagree with each statement on a 7-point Likert scale ranging from 1 (strongly disagree) to 7 (strongly agree). The present study’s Cronbach’s alpha value was 0.89 for the SWLS.

##### Positive affect and negative affect

Positive affect and negative affect were assessed using the Scale of Positive and Negative Experience (SPANE). The validity and reliability of SPANE have been tested previously among Chinese college students (22). The SPANE has 12 items describing positive and negative feelings. The respondents were asked to rate the extent to which they experienced each item using a 5-point Likert scale from 1 (very rarely or never) to 5 (very often or always). The Cronbach’s alpha values were 0.92 for positive affect and 0.90 for negative affect in the current study.

#### 2.3.2 The inflammatory biomarkers

To estimate the effect of different exercise intervention modes on the inflammatory factors and their association with psychometric scales. We assessed the inflammation biomarkers, including immunoglobulin A (IgA), immunoglobulin M (IgM), albumin (Alb), globulin (GLO), lymphocytes (LYM), and lymphocyte percentage (LYM), which were assessed by DxC 800 Chemistry Analyzer (Beckman Coulter, USA). The kit was developed by Leadman Biochemical Company (Beijing, China).

### 2.4 Statistical analysis

We firstly performed descriptive analysis to obtain frequency and percentage for categorical variables and mean and standard deviation (SD) for continuous variables. Differences between groups at baseline were determined using a one-way analysis of variance (ANOVA) for continuous variables and a chi-square test for categorical variables, respectively. Considering the repeated measurement study design, we adopted the mixed-effect model with a random effect on individuals nested within the group to account for intrapersonal variation [R-packages: lme4 and nlme4 (29)]. The mixed-effect model in our study included the fixed effect of group (control, MVCT, and HIIT), measurement time (pre- and post-test), and their interaction, and controlled for fixed effects of other covariates, including gender, age, education level, residence, self-perceived income level, and body mass index (BMI). The interaction term between the group and measurement time estimated change in the dependent before and after the intervention by group, representing the causal effect of exercise intervention. Our mixed-effect model used maximum likelihood estimation and a Kenward Rogers adjustment to the degrees of freedom for an intent-to-treat analysis. Pairwise comparisons of changing health outcomes between groups during the intervention were further performed [R-packages: emmeans (30)]. Moreover, linear regression models were used to assess the correlations between the change in the inflammation markers and the change in mental health variables adjusted for gender, age, education level, residence, self-perceived income level, and BMI. All data analysis and visualization were conducted via R software (version 4.1.2), and *p*-value less than 0.05 were considered statistically significant.

## 3 Results

### 3.1 Demographic information at baseline

As is shown in Table 2, male participants accounted for approximately half, and the average age was 25.26 ± 2.21 years. More than 70% of the participants were urban residents and reported lower self-perceived income levels. Similar baseline characteristics between the three groups were observed except for Alb and Glo, with values in the MVCT group being significantly higher than those in the other two groups (Table 2).

**TABLE 2:** Baseline characteristics of the participants.

Variables	Control	MVCT	HIIT	Total	*p*
		
	*N* = 28	*N* = 33	*N* = 32	*N* = 93	
**Categorical variables, N (%)**					
Male	13 (46.43)	16 (48.48)	14 (43.75)	43 (46.24)	0.93
Whether urban residence (yes)	17 (60.71)	27 (81.82)	27 (84.38)	71 (76.34)	0.065
Self-perceived income level (lower)	21 (75.00)	29 (87.88)	27 (84.38)	77 (82.80)	0.60
**Continuous variables, mean (SD)**					
Age (years)	25.39 ± 2.64	25.42 ± 1.56	24.97 ± 2.39	25.26 ± 2.21	0.66
Received higher education (years)	7.07 ± 2.45	6.76 ± 1.35	6.16 ± 1.46	6.65 ± 1.80	0.13
Body mass index (kg/m^2^)	21.52 ± 2.87	22.56 ± 3.30	21.99 ± 2.57	22.05 ± 2.93	0.38
Body fat ratio (%)	27.27 ± 5.20	25.79 ± 5.58	24.82 ± 3.98	25.91 ± 5.00	0.18
VO_2max_ [mL/(kg⋅min)]	44.92 (7.01)	42.31 (6.31)	43.90 (7.63)	43.71 (7.26)	0.37
Satisfaction score	4.84 ± 0.95	4.42 ± 0.74	4.72 ± 1.03	4.65 ± 0.92	0.19
Positive score	4.27 ± 1.07	4.32 ± 0.80	4.65 ± 0.90	4.42 ± 0.93	0.23
Negative score	2.83 ± 1.14	2.98 ± 1.00	2.81 ± 1.00	2.88 ± 1.04	0.76
Well-being score	6.28 ± 2.43	5.76 ± 2.07	6.57 ± 2.10	6.20 ± 2.20	0.33
IgA (g/L)	2.20 ± 0.65	2.40 ± 0.66	2.33 ± 0.69	2.32 ± 0.66	0.53
IgM (g/L)	1.45 ± 0.60	1.57 ± 0.42	1.45 ± 0.71	1.49 ± 0.58	0.67
Alb (g/L)	53.08 ± 1.76	58.50 ± 2.70	56.49 ± 2.49	56.22 ± 3.22	<**0.001**
Glo (g/L)	22.89 ± 3.04	29.54 ± 3.68	26.53 ± 3.27	26.56 ± 4.27	<**0.001**
LYM	2.10 ± 0.67	2.08 ± 0.51	2.06 ± 0.43	2.08 ± 0.54	0.96
LYM%	37.36 ± 7.58	35.92 ± 7.64	36.99 ± 7.89	36.72 ± 7.65	0.75

SD, standard deviation; IgA, Immunoglobulin A; IgM, Immunoglobulin M; Alb, Albumin; Glo, Globulin; LYM, absolute value of lymphocyte count; LYM%, the ratio of lymphocyte to total leukocyte. Well-being = satisfaction + positive affect – negative affect. P-values are differences between three groups under the chi-square test (categorical variables) or one-way ANOVA (continuous variables). Statistically significant results at 0.05 level are presented in bold.

### 3.2 Effects of the intervention on mental health indicators

**FIGURE 3:**
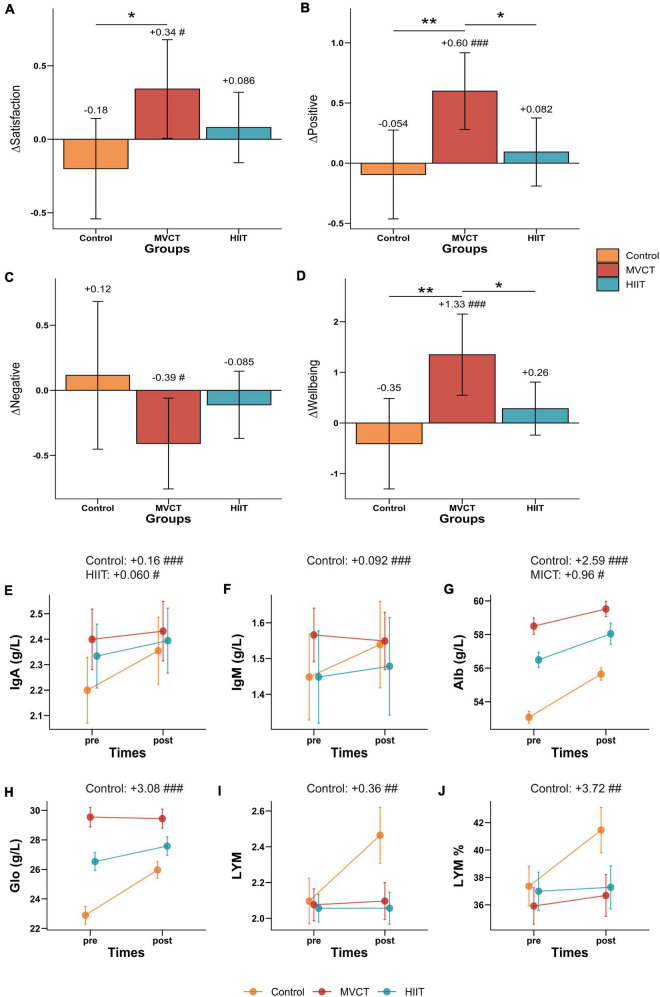
Mental health and blood biochemical indicators measured in pre-post three-month intervention project. MVCT, moderate-to-vigorous intensity continuous training; HIIT, high-intensity interval training. IgA, Immunoglobulin A; IgM, Immunoglobulin M; Alb, Albumin; Glo, Globulin; LYM, absolute value of lymphocyte count; LYM%, the ratio of lymphocyte to total leukocyte. Subgraphs **(A–D)** show changing values of four mental health indicators during the intervention period, and error bars indicate a 95% confidence interval. Subgraphs **(E–J)** show the absolute mean value of six biochemical indicators at two measurement time points, and error bars indicate standard error. Symbol of # indicates the statistically significant difference of the certain group from pre to post-measurement; * indicates the statistically significant difference between groups during the pre-post intervention period. Results are calculated using mixed-effect models adjusted for gender, age, education level, residence, income level, and body mass index with a random effect on individuals to account for intrapersonal variation. *p < 0.05; **p < 0.01; #*p* < 0.05; ##*p* < 0.01; ###*p* < 0.001.

Significant upward trends during the three-month intervention period were observed in the MVCT group for satisfaction [adjusted mean difference (ΔMean) = 0.34, *p* = 0.021] positive affect (ΔMean = 0.60, *p* < 0.001), and well-being (ΔMean = 1.33, *p* < 0.001), and a significant downward trend for negative affect (ΔMean = −0.39, *p* = 0.039) were also found in this group (Figure 3 and Supplementary Table 1). Similar to the MVCT group, changing tendency for these four mental health outcomes were observed in the HIIT group, while totally opposite trends of those occurred in the control group, even if those changes in the HIIT and control group was not significant (Figure 3 and Supplementary Table 1). Moreover, the statistically significant difference in change of satisfaction (β [95% CI]: 0.52 [0.10, 0.93], *p* = 0.018), positive affect (0.66 [0.22, 1.09], *p* = 0.0041), and well-being (1.68 [0.67, 2.68], *p* = 0.0017) between the MVCT and control group was observed after the intervention, but that was not found between the HIIT and control group (Table 3 and Supplementary Table 2). Also, the MVCT group increased significantly larger in positive affect (0.52 [0.10, 0.94], *p* = 0.018) and well-being (1.08 [0.099, 2.05], *p* = 0.035) than those in the HIIT group (Table 3 and Supplementary Table 2).

**TABLE 3:** Results of mixed-effect models: Differences between groups of changes in mental health and blood biochemical indicators from pre to post measurement^a^.

Variable	Differences, post vs. pre, MVCT vs. Control		Difference, post vs. pre, HIIT vs. Control		Difference, post vs. pre, MVCT vs. HIIT	
	β (95% CI)	*p*	β (95% CI)	*p*	β (95% CI)	*p*
Satisfaction	0.52 (0.10, 0.93)	**0.018**	0.26 (−0.16, 0.68)	0.23	0.25 (−0.15, 0.66)	0.22
Positive	0.66 (0.22, 1.09)	**0.0041**	0.14 (−0.30, 0.57)	0.55	0.52 (0.10, 0.94)	**0.018**
Negative	−0.51 (−1.04, 0.029)	0.069	−0.20 (−0.74, 0.34)	0.47	−0.30 (−0.82, 0.21)	0.26
Well−being	1.68 (0.67, 2.68)	**0.0017**	0.61 (−0.42, 1.62)	0.25	1.08 (0.099, 2.05)	**0.035**
IgA (g/L)	−0.14 (−0.21, −0.067)	<**0.001**	−0.096 (−0.17, −0.021)	**0.016**	−0.045 (−0.12, 0.027)	0.23
IgM (g/L)	−0.12 (−0.19, −0.056)	<**0.001**	−0.068 (−0.13, −0.0021)	**0.048**	−0.053 (−0.12, 0.010)	0.11
Alb (g/L)	−1.64 (−2.96, −0.30)	**0.019**	−1.14 (−2.48, 0.20)	0.10	−0.50 (−1.77, 0.78)	0.45
Glo (g/L)	−3.17 (−4.67, −1.68)	<**0.001**	−2.07 (−3.57, −0.56)	**0.0093**	−1.11 (−2.54, 0.33)	0.14
LYM	−0.34 (−0.65, −0.035)	**0.034**	−0.35 (−0.66, −0.038)	**0.032**	0.0065 (−0.29, 0.30)	0.97
LYM%	−2.77 (−6.17, 0.60)	0.12	−3.97 (−7.36, −0.55)	**0.027**	1.20 (−2.08, 4.43)	0.48

β, interactive effect estimates of the mixed-effect model, indicating the between-group difference during the three-month intervention period; CI, confidence interval. MVCT, moderate-to-vigorous intensity continuous training; HIIT, high-intensity interval training. IgA, Immunoglobulin A; IgM, Immunoglobulin M; Alb, Albumin; Glo, Globulin; LYM, absolute value of lymphocyte count; LYM%, the ratio of lymphocyte to total leukocyte. ^a^Differences between groups in change from pre to post-test, calculated via mixed-effect models adjusted for gender, age, education level, residence, income level, and body mass index, with a random effect on individuals to account for intrapersonal variation. For instance, “Differences, post vs. pre, MVCT vs. Control” indicates the calculation formula that (post_MVCT_ – pre_MVCT_) – (post_Control_ – pre_Control_). Statistically significant results at 0.05 level are presented in bold.

### 3.3 Effects of the intervention on inflammatory factors outcomes

We found that blood inflammation factors in the control group significantly increased by 0.16 g/L, 0.092 g/L, 2.59 g/L, 3.08 g/L, 0.36, and 3.72% in IgA (*p* < 0.001), IgM (*p* < 0.001), Alb (*p* < 0.001), Glo (*p* < 0.001), LYM (*p* = 0.0027), and LYM percent (*p* = 0.0045), respectively, indicating an increase of inflammation in blood in the control group (Figure 3 and Supplementary Table 1). Significant differences in the change of IgA, IgM, Glo, and LYM were found in HIIT and MVCT groups compared with the control group (e.g., for IgA, MVCT vs. control: −0.14 [−0.21, −0.067], *p* < 0.001; HIIT vs. control: −0.096 [−0.17, −0.021], *p* = 0.016) (Table 3 and Supplementary Table 2). The HIIT and MVCT group also showed a similar trend in these inflammation outcomes, with those in the HIIT group increasing slightly more than those in the MVCT group overall (Figure 3). However, in terms of immune-inflammatory outcomes, no significant change in the HIIT and MVCT groups was observed (Table 3 and Supplementary Table 2).

### 3.4 Correlations between the changing inflammatory factors and mental health variables

**FIGURE 4:**
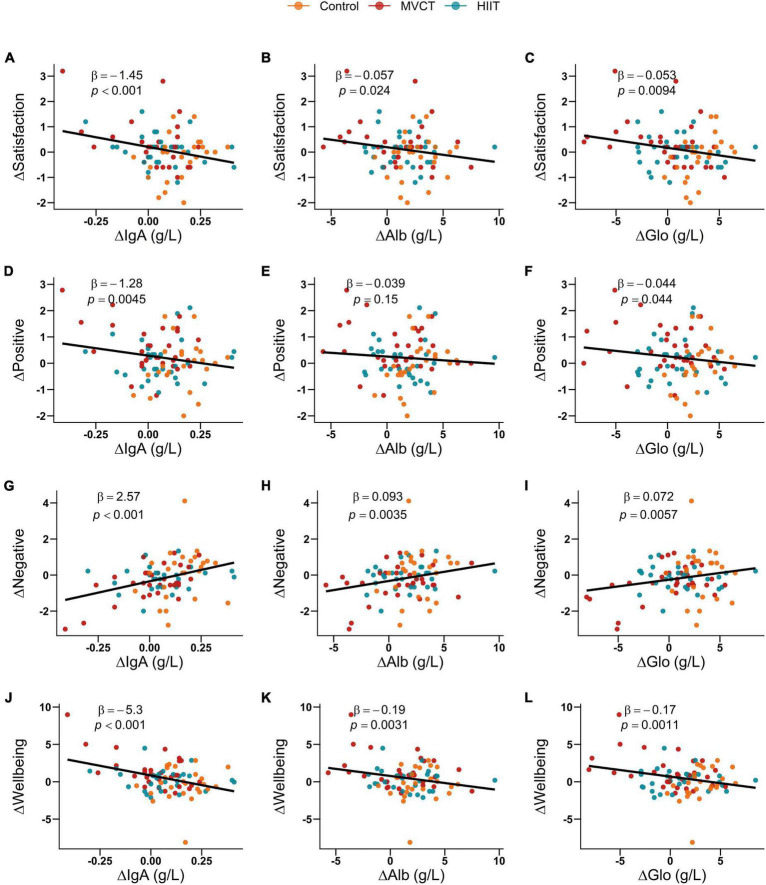
Correlations between the changing blood biochemical indicators and mental health outcomes. MVCT, moderate-to-vigorous intensity continuous training; HIIT, high-intensity interval training. IgA, Immunoglobulin A; Alb, Albumin; Glo, Globulin. β, effect estimate of linear regression model adjusted for gender, age, education level, residence, income level, and body mass index. Subgraphs **(A–C)** indicate the correlations between ΔIgA, ΔAlb, ΔGlo, and Δsatisfaction, respectively. Subgraphs **(D–F)** indicate the correlations between ΔIgA, ΔAlb, ΔGlo, and Δpositive, respectively. Subgraphs **(G–I)** indicate the correlations between ΔIgA, ΔAlb, ΔGlo, and Δnegative, respectively. Subgraphs **(J–L)** indicate the correlations between ΔIgA, ΔAlb, ΔGlo, and Δwell-being, respectively.

According to the adjusted linear regression model, associations of ΔIgA, ΔAlb, and ΔGlo with satisfaction, negative affect, and well-being were statistically significant. Furthermore, an increase of 1 g/L in ΔIgA (*p* = 0.0045) and ΔGlo (*p* = 0.044) was significantly associated with a decrease of 1.28 and 0.044 scores in positive affect, and an increase of 1 g/L in ΔIgM was significantly correlated with an increase of 1.30 scores in negative affect (*p* = 0.034) (Figure 4 and Supplementary Table 3). However, LYM and LYM% were not significantly correlated with mental health outcomes.

## 4 Discussion

In the 12-week double-blinded RCT, we explored which exercise intervention mode is more suitable for preventing mental health problems and the potential mechanisms of the inflammation pathway. Firstly, we found that mental health problems and inflammation factors increased in the control group during 12 weeks, demonstrating how quickly mental problems can surge for college students especially in examination weeks during their academic term. Secondly, the 12-week MVCT intervention was effective in defending against the increased inflammatory response and could improve mental health, while HIIT intervention significantly reduced inflammatory factors and resulted in a positive but insignificant trend in mental health and well-being, indicating that the MVCT modality was more conducive to enhance positive affect and well-being than the HIIT. Moreover, we found that IgA, Alb, and Glo, were significantly associated with well-being, validating one of the mechanisms in young adults that exercise improved mental health via the potential pathway of the immune system. Thus, this study indicates that both MVCT and HIIT are beneficial to the immune system, and MVCT is more recommended to promote mental health. Our findings provide meaningful strategies for healthcare workers.

We investigated the college students’ mental health and well-being at the beginning and repeated the measurement at the end of their academic term. This group of college students is known to live under high pressure, especially in the second measurement during the exam weeks. Moreover, all the college students stayed on campus all semester due to social distancing during COVID-19, which would greatly reduce the frequency of their social and entertainment activities outside the school. As a result, they were more vulnerable to accumulating negative emotions like stress and depression (31). Our findings are consistent with these interpretations and events. After just 12 weeks, the non-exercise control group represented a significantly negative trend in not only immune inflammation but also mental health status, indicating that college students may be confronted with a rapid decline in mental health under persistently high levels of perceived stress.

We wondered if exercise improved mental health, especially for college students. As expected, exercise intervention could effectively increase satisfaction, positive affect, and well-being and reduce college students’ adverse effects to some extent. This finding is consistent with previous observational studies among healthy adults and mental disorder patients (32–34). A cross-sectional study that included 1.2 million people over 18 years in the USA indicated that individuals who exercised had 1.49 fewer days of poor mental health in the past month than those who did not exercise. Popular team sports, aerobic activities, durations of 45 min, and frequencies of three to five times per week were recommended for optimum mental health (32). These studies indicate that exercise, especially aerobic exercise, can protect the mental health of people of all ages and those who are vulnerable. Also, some RCT studies represented similar findings among older people and depressive patients. An 8-week intervention study illustrated that treadmill walking exercise training was an effective strategy to improve quality of life and psychological well-being in Alzheimer elderly subjects (33); a 12-week aerobic training study indicated that no matter whether light, moderate or vigorous exercise helps treat mild to moderate depressive symptoms like a physician (34).

A critical question we sought to address was whether different exercise modalities had the same impact on mental health and shared a similar inflammatory mechanism. Indeed, we found that both 12-week MVCT and HIIT could prevent inflammatory response without significant differences. However, the MVCT intervention modality was more effective in improving mental health and well-being for college students than HIIT. These findings suggest that MVCT works better than HIIT to improve mental health status, which was associated with the decrease of biochemical markers related to inflammation, such as IgA, Alb, and LYM. This result was consistent with a 6-week RCT study conducted on Canadian university students, indicating that although HIIT decreased depressive symptoms, it also increased perceived stress, TNF-α and IL-6 compared to MVCT, and MVCT may be the optimal exercise modality to promote mental health (35). Similarly, a 16-week high-intensity functional exercise program indicated no superior effect on depressive symptoms relative to the non-exercise control group (36).

According to the biochemical indicators, both MVCT and HIIT exercises had anti-inflammatory effects. Consistent with our findings, previous studies also documented similar results that aerobic exercise is effective in reducing depression symptoms via declining systemic inflammation [e.g., tumor necrosis factor-alpha (TNF-α), interleukin-6 (IL-6), and C-reactive protein (CRP)] in patients with chronic obstructive pulmonary disease (37). A 12-week intervention study documented that exercise could decrease the circulation of IL-6 in individuals with type 2 diabetes (38); 14-week aerobic exercise effectively reduced CRP by 15% among female patients with diabetes (39). Furthermore, previous studies found that regular aerobic exercise correlated with a lower CRP and IL-6 in the elderly (40). The benefit of exercise on well-being and satisfaction could be attributable to the mechanisms of neuroendocrine system (20), oxidative stress (41), and inflammation (42). Firstly, exercise could change the homeostasis of blood glucose, resulting in stimulation of the HPA axis, and thus cortisol secretion, which was proved to be associated with life satisfaction (43). Moreover, BDNF, GABAergic system, and endocannabinoid system are involved in the stress-mediated responses, which play an important role in the mechanism of exercise and homeostatic stress. Secondly, a previous study revealed that three weeks of exercise is sufficient to begin promoting alterations in the oxidative stress system and improve the depression status (41), which was proved to be strongly associated with life satisfaction in the 15-year follow-up of healthy adults (44). The conclusion also proved in the animal model that exercise prevented an increase in the thiobarbituric acid-reactive substances serum levels in hyperphenylalaninemic rats (45). Thirdly, the possible biological mechanisms underlying such anti-inflammatory effects of exercise including reduced visceral fat mass (46), increased release of anti-inflammatory cytokines from contracting skeletal muscle (47), and declined expression of Toll-like receptors (TLRs) on monocytes and macrophages, which can subsequently inhibit the production of pro-inflammation (48). Our finding confirmed and expanded the results of a few studies through randomized controlled trials, enriching inflammation as a potentially crucial biological pathway for exercise to improve life satisfaction.

Interestingly, we found associations between the immune system and the level of satisfaction in this study. This finding is consistent with the evidence that C-Reactive protein was also associated with satisfaction after accounting for the health behaviors (28). satisfaction is an important causal factor to immune system and eventually affects health (49). On the other hand, it may also be a reverse causal relationship. The decrease of inflammation leads to an increase in satisfaction. Individual differences in life satisfaction are largely due to genetic factors, and inflammation and life satisfaction may have shared genetic factors, such as serotonin transportation (50, 51). Wellbeing (life satisfaction and happiness) was reported to associate with methylation of the same promotors, such as intercellular adhesion molecule 1 (ICAM-1) and Tissue Factor (F3) in leukocytes (52), both of which are involved in inflammatory processes. In fact, excessive exercise intensity may not always be better (32). Evidence showed that high-intensity exercise could increase perceived stress regulated by the hypothalamic-pituitary axis (HPA) axis (53, 54). Hence, we hypothesized that the physiological response to high-intensity exercise might exacerbate the physiological response to psychological stressors and thus decrease the positive effect on mental health. Future research needs to discuss the best does and loads of high-intensity of exercise for reducing stress and improving mental health.

There are several limitations to this study. Firstly, we measured some of the inflammatory factors of the peripheral blood, and further studies are needed to explore the role of other inflammatory factors, such as TNF-α, IL-6, and IL-12. Moreover, the changes in the concentration of endocannabinoids in serum after exercise intervention also need further discussion. Secondly, we conducted the exercise intervention for 12 weeks in this study. We found the positive effect of MVCT on mental health, but we did not stratify the dose of HIIT and MVCT intervention load. Future research needs to explore the impact and optimum exercise doses of different intervention modalities on mental health. Thirdly, participants were asked not to eat after 8:00 pm on the day before the test and to take peripheral blood 12 h after fasting. However, the amount of drinking water may impact inflammatory indicators. Thus, water consumption should be controlled in future studies. However, this study also has notable strengths. Firstly, to the best of our knowledge, the double-blinded RCT study provided first-hand evidence that compared the effect of HIIT and MVCT interventions on mental health among young adults. Also, the intensity of MVCT and HIIT was determined by the individual maximal oxygen uptake obtained from a laboratory cardiopulmonary exercise test and would be more accurate than other studies. Most importantly, this RCT study provides valuable insights into the causal effect of HIIT/MVCT interventions on mental health and its potential mechanism of inflammatory factors.

## 5 Conclusion

In summary, our study suggests that both 12-week MVCT and HIIT interventions benefit the immune system, and MVCT is more recommended to promote mental health. This double-blinded RCT also proves one of the potential mechanisms that exercise improves mental health through the pathway of blood inflammation factors. The study could provide first-hand evidence of the effect of 12-week exercises on mental health among young adults, enlighten practical implications for public health to prevent mental disorders, and inspire ideas for treating other chronic inflammatory diseases.

## Data availability statement

The original contributions presented in this study are included in the article/Supplementary material, further inquiries can be directed to the corresponding authors.

## Ethics statement

The studies involving human participants were reviewed and approved by the Academic Ethics Committee of Tsinghua University. The patients/participants provided their written informed consent to participate in this study.

## Author contributions

XM and JL contributed to the conception and design of the study. XL and DW collected the blood sample. YY and LM collected the mental health data. BL performed the intervention of different groups. BS and YL processed the blood sample. JL wrote and completed the revision of the manuscript. YZ completed the data curation and wrote the discussion part. QD and XM revised and edited the manuscript. QD, XM, and JL provided the supporting funding. All authors were involved in the experiment, critical review of the manuscript, and approved the final version.
