# Supplementary material for: Exercise improves mental health status of young adults via attenuating inflammation factors but modalities matter
Source: Front Psychiatry. 2022 Dec 14;13:1067890. doi: 10.3389/fpsyt.2022.1067890 (PMC9795189; doi:10.3389/fpsyt.2022.1067890)
Supplement: Supplementary file 1 [file Data_Sheet_1.docx]

**Exercise Improves Mental Health Status of Young Adults via Attenuating Inflammation Factors but Modalities Matter**

**Table S1. Adjusted mean (95% CI) values of mental health and blood biochemical indicators among three groups at two measurement time points.**

| **Variables** | **Group** | **Pre-test**  **Adjusted mean (95% CI)** | **Post-test**  **Adjusted mean (95% CI)** | **Adjusted mean difference**  **Δ (Post–Pre)** | ***p*** |
| --- | --- | --- | --- | --- | --- |
| **Satisfaction** | Control | 4.82 (4.44, 5.21) | 4.65 (4.25, 5.04) | -0.18 | 0.26 |
|  | MVCT | 4.47 (4.09, 4.84) | 4.81 (4.43, 5.19) | 0.34 | **0.021** |
|  | HIIT | 4.75 (4.35, 5.14) | 4.83 (4.43, 5.23) | 0.086 | 0.56 |
| **Positive** | Control | 4.28 (3.94, 4.62) | 4.23 (3.88, 4.57) | -0.054 | 0.74 |
|  | MVCT | 4.44 (4.11, 4.77) | 5.05 (4.71, 5.38) | 0.60 | **< 0.001** |
|  | HIIT | 4.76 (4.42, 5.11) | 4.85 (4.50, 5.20) | 0.082 | 0.60 |
| **Negative** | Control | 2.81 (2.39, 3.22) | 2.92 (2.50, 3.35) | 0.12 | 0.56 |
|  | MVCT | 2.99 (2.58, 3.39) | 2.60 (2.19, 3.01) | -0.39 | **0.039** |
|  | HIIT | 2.81 (2.40, 3.23) | 2.73 (2.30, 3.16) | -0.085 | 0.66 |
| **Wellbeing** | Control | 6.30 (5.45, 7.15) | 5.95 (5.08, 6.82) | -0.35 | 0.36 |
|  | MVCT | 5.92 (5.09, 6.75) | 7.26 (6.41, 8.10) | 1.33 | **< 0.001** |
|  | HIIT | 6.69 (5.83, 7.56) | 6.95 (6.07 7.83) | 0.26 | 0.48 |
| **IgA (g/L)** | Control | 2.16 (1.88, 2.44) | 2.32 (2.03, 2.60) | 0.16 | **< 0.001** |
|  | MVCT | 2.36 (2.09, 2.63) | 2.37 (2.10, 2.64) | 0.015 | 0.57 |
|  | HIIT | 2.33 (2.05, 2.61) | 2.39 (2.11, 2.67) | 0.060 | **0.027** |
| **IgM (g/L)** | Control | 1.42 (1.19, 1.65) | 1.51 (1.28, 1.74) | 0.092 | **< 0.001** |
|  | MVCT | 1.61 (1.40, 1.83) | 1.59 (1.37, 1.80) | -0.029 | 0.20 |
|  | HIIT | 1.49 (1.26, 1.72) | 1.51 (1.28, 1.74) | 0.024 | 0.31 |
| **Alb (g/L)** | Control | 53.1 (52.1, 54.1) | 55.7 (54.7, 56.6) | 2.59 | **< 0.001** |
|  | MVCT | 58.7 (57.8, 59.6) | 59.6 (58.7, 60.6) | 0.96 | 0.041 |
|  | HIIT | 56.7 (55.8, 57.7) | 58.2 (57.2, 59.1) | 1.45 | 0.0024 |
| **Glo (g/L)** | Control | 22.8 (21.4, 24.1) | 25.8 (24.5, 27.2) | 3.08 | **< 0.001** |
|  | MVCT | 29.7 (28.4, 31.0) | 29.6 (28.3, 30.9) | -0.098 | 0.85 |
|  | HIIT | 26.7 (25.4, 28.1) | 27.7 (26.4, 29.1) | 1.01 | 0.058 |
| **LYM** | Control | 2.09 (1.87, 2.31) | 2.45 (2.22, 2.69) | 0.36 | **0.0027** |
|  | MVCT | 2.06 (1.84, 2.28) | 2.08 (1.85, 2.30) | 0.021 | 0.85 |
|  | HIIT | 2.05 (1.82, 2.27) | 2.06 (1.83, 2.29) | 0.014 | 0.90 |
| **LYM %** | Control | 37.1 (33.9, 40.3) | 40.8 (37.6, 44.1) | 3.72 | **0.0045** |
|  | MVCT | 35.7 (32.6, 38.8) | 36.7 (33.5, 39.8) | 0.96 | 0.42 |
|  | HIIT | 36.6 (33.3, 39.8) | 36.3 (33.0, 39.6) | -0.25 | 0.84 |

Note: CI, confidence interval; MVCT, moderate-to-vigorous intensity continuous training; HIIT, high intensity interval training. IgA, Immunoglobulin A; IgM, Immunoglobulin M; Alb, Albumin; Glo, Globulin; LYM, absolute value of lymphocyte count; LYM %, ratio of lymphocyte to total leukocyte. Results are calculated using mixed effect models adjusted for gender, age, education level, residence, income level and body mass index with a random effect on individuals to account for intrapersonal variation. Statistically significant results at 0.05 level are presented in bold.

**Table S2. Results of mixed effect models: Changes in mental health and blood biochemical indicators from pre to post measurement.**

| **Variable** | **Group ^a^**  **β (95% CI)** | | **Time ^b^**  **β (95% CI)** | **Group*Time ^c^**  **β (95% CI)** | |
| --- | --- | --- | --- | --- | --- |
|  | MICT | HIIT |  | MICT*post | HIIT*post |
| **Satisfaction** | -0.36 (-0.86, 0.15) | -0.079 (-0.60, 0.44) | -0.18 (-0.48, 0.13) | 0.52 (0.10, 0.93) * | 0.26 (-0.16, 0.68) |
| **Positive** | 0.16 (-0.28, 0.60) | 0.48 (0.027, 0.94) * | -0.053 (-0.37, 0.27) | 0.66 (0.22, 1.09) ** | 0.14 (-0.30, 0.57) |
| **Negative** | 0.18 (-0.36, 0.72) | 0.0096 (-0.55, 0.57) | 0.12 (-0.28, 0.51) | -0.51 (-1.04, 0.029) | -0.20 (-0.74, 0.34) |
| **Wellbeing** | -0.38 (-1.49, 0.73) | 0.39 (-0.75, 1.54) | -0.35 (-1.09, 0.40) | 1.68 (0.67, 2.68) ** | 0.61 (-0.42, 1.62) |
| **IgA (g/L)** | 0.20 (-0.16, 0.55) | 0.17 (-0.20, 0.54) | 0.16 (0.10, 0.21) *** | -0.14 (-0.21, -0.067) *** | -0.096 (-0.17, -0.021) * |
| **IgM (g/L)** | 0.20 (-0.093, 0.49) | 0.069 (-0.23, 0.37) | 0.092 (0.044, 0.14) *** | -0.12 (-0.19, -0.056) *** | -0.068 (-0.13, -0.0021) * |
| **Alb (g/L)** | 5.59 (4.36, 6.83) *** | 3.65 (2.38, 4.92) *** | 2.59 (1.61, 3.58) *** | -1.64 (-2.96, -0.30) * | -1.14 (-2.48, 0.20) |
| **Glo (g/L)** | 6.97 (5.22, 8.71) *** | 3.95 (2.14, 5.76) *** | 3.08 (1.97, 4.18) *** | -3.17 (-4.67, -1.68) *** | -2.07 (-3.57, -0.56)  ** |
| **LYM** | -0.032 (-0.33, 0.26) | -0.044 (-0.35, 0.26) | 0.36 (0.14, 0.59) ** | -0.34 (-0.65, -0.035) * | -0.35 (-0.66, -0.038) * |
| **LYM %** | -1.41 (-5.58, 2.74) | -0.54 (-4.84, 3.76) | 3.72 (1.24, 6.23) ** | -2.77 (-6.17, 0.60) | -3.97 (-7.36, -0.55) * |

Note: β, effect estimate; CI, confidence interval; Wellbeing = satisfaction + positive affect – negative affect. MVCT, moderate-to-vigorous intensity continuous training; HIIT, high-intensity interval training. IgA, Immunoglobulin A; IgM, Immunoglobulin M; Alb, Albumin; Glo, Globulin; LYM, absolute value of lymphocyte count; LYM %, the ratio of lymphocyte to total leukocyte. Results are calculated using mixed effect models adjusted for gender, age, education level, residence, income level, and body mass index with a random effect on individuals to account for intrapersonal variation.

^a^ Reference category: Control group

^b^ Reference category: Pre measurement

^c^ Differences between the MICT and control group or the HIIT and control group from pre- to post-measurement, indicating the intervention effect.

* *p* < 0.05, ** *p* < 0.01, *** *p* < 0.001

**Table S3. Correlations between the changing blood biochemical indicators and mental health outcomes.**

| Biochemical variables | Satisfaction | | Positive | | Negative | | Wellbeing | |
| --- | --- | --- | --- | --- | --- | --- | --- | --- |
|  | β | P | β | P | β | P | β | P |
| IgA (g/L) | -1.45 | **< 0.001** | -1.28 | **0.0045** | 2.57 | **< 0.001** | **-**5.30 | **< 0.001** |
| IgM (g/L) | -0.13 | 0.79 | -0.49 | 0.34 | 1.30 | **0.034** | -1.92 | 0.12 |
| Alb (g/L) | -0.057 | **0.024** | -0.039 | 0.15 | 0.093 | **0.0035** | -0.19 | **0.0031** |
| Glo (g/L) | -0.053 | **0.0094** | -0.044 | **0.044** | 0.072 | **0.0057** | -0.17 | **0.0011** |
| LYM | -0.17 | 0.13 | -0.11 | 0.36 | -0.030 | 0.83 | -0.25 | 0.38 |
| LYM % | -0.010 | 0.31 | 0.017 | 0.11 | -0.015 | 0.23 | 0.022 | 0.39 |

Note: IgA, Immunoglobulin A; IgM, Immunoglobulin M; Alb, Albumin; Glo, Globulin; LYM, absolute value of lymphocyte count; LYM %, the ratio of lymphocyte to total leukocyte. β, effect estimate of linear regression model adjusted for gender, age, education level, residence, income level and body mass index. Statistically significant results at 0.05 level are presented in bold.

**Table S4. The running distance and** **lactic acid change after one session of the interventions**

| **Group** | **Running distance of one session** | **Lactic acid** | |
| --- | --- | --- | --- |
|  |  | **Baseline** | **Post-test of one session** |
| MVCT (N=33) | 2.68±0.35 | 1.27±0.86 | 4.63±1.84 |
| HIIT (N=32) | 2.66±0.29 | 1.36±0.77 | 5.76±2.36 |
| Control (28) | / | 1.09±0.73 | 2.64±1.18 |
| *t (F)* | 0.279 | 6.999 | |
| *P* | 0.781 | < 0.001 | |

Note: The running distance and the lactic acid were measured in the first session of the intervention. MVCT, moderate-to-vigorous intensity continuous training; HIIT, high-intensity interval training.

**Table S5. The coefficient of variations (CVs) of the blood parameters.**

| Biochemical variables | CV | |
| --- | --- | --- |
|  | Time1 | Time3 |
| IgA (g/L) | 28.82% | 27.69% |
| IgM (g/L) | 38.93% | 39.77% |
| Alb (g/L) | 5.66% | 5.36% |
| Glo (g/L) | 16.18% | 12.70% |
| LYM | 25.98% | 28.99% |
| LYM % | 21.07% | 22.51% |

Note: IgA, Immunoglobulin A; IgM, Immunoglobulin M; Alb, Albumin; Glo, Globulin; LYM, absolute value of lymphocyte count; LYM %, the ratio of lymphocyte to total leukocyte. CVs = (SD/Mean) *100%.

**Table S6. The parameters during the VO_2max_ test in the baseline.**

| **Variables** | **MVCT** | **HIIT** | **Control** | ***F*** | ***p*** |
| --- | --- | --- | --- | --- | --- |
|  | N = 33 | N = 32 | N = 28 |  |  |
| Heart rate | 182.00 | 184.68 | 189.009 | *2.99* | *> 0.05* |
| Minute ventilation (L/min) | 94.79 | 97.3992 | 104.66 | *0.78* | *> 0.05* |
| VE/VO_2_ | 33.13 | 32.36 | 35.19 | *1.48* | *> 0.05* |
| VE/VCO_2_ | 28.54 | 29.01 | 30.62 | 1.77 | *> 0.05* |

Note: VE/VO_2_ (ventilatory equivalent for oxygen), the ratio of the minute ventilation (VE) and the volume of oxygen consumed; VE/VO_2_ (ventilation equivalent for carbon dioxide), the ratio of minute ventilation (VE) and the volume of carbon dioxide production (VCO_2_)；MVCT, moderate-to-vigorous intensity continuous training; HIIT, high-intensity interval training. One-way ANOVA was used to estimate the difference between the groups on the outcomes in the baseline
